# Characterization of the complete chloroplast genome of a subalpine deciduous shrub *Lonicera angustifolia* var. *myrtillus* (Caprifoliaceae)

**DOI:** 10.1080/23802359.2021.1931507

**Published:** 2021-05-31

**Authors:** Yuan-Mi Wu, Di Ma, Yi-Xuan Zhu, Bing Liu, Xian-Yun Mu

**Affiliations:** a Laboratory of Systematic Evolution and Biogeography of Woody Plants, School of Ecology and Nature Conservation, Beijing Forestry University, Beijing, China; b State Key Laboratory of Systematic and Evolutionary Botany, Institute of Botany, Chinese Academy of Sciences, Beijing, China

**Keywords:** Complete chloroplast genome, phylogeny, Caprifoliaceae, *Lonicera*

## Abstract

The genus *Lonicera* (Caprifoliaceae) is of great economical significance. It has been taxonomically studied frequently in history, while phylogenetic relationships intra the genus are still obscure. Here, we reported the first species complete chloroplast genome sequence in the section *Isoxylosteum*, *Lonicera angustifolia* var. *myrtillus*. It is 156,222 bp in length, comprising a large single-copy (LSC) region of 89,838 bp, a small single-copy (SSC) region of 19,211 bp, and a pair of inverted repeats (IRs) of 23,509 bp. In *L. angustifolia* var. *myrtillus* chloroplast genome, a total of 114 functional genes were identified, with an overall GC content of 38.4%. The phylogenetic relationships of *Lonicera* based on maximum-likelihood (ML) showed that *L. angustifolia* var. *myrtillus* is most closely related to *L. nervosa* in section *Isika*. Our study contributes to the molecular phylogenetic studies of *Lonicera* and Caprifoliaceae.

The genus *Lonicera* (Caprifoliaceae) have significant ornamental, medicinal, and edible value. There are approximately 200 species in *Lonicera* which are mainly distributed in temperate and subtropical areas in the Northern Hemisphere, with several species extending their range into tropical areas of India, Malaysia, Philippines, and North Africa (Rehder 1903, 1913; van Steenis 1946; Theis et al. 2008), some of them are highly endangered, such as *L. oblata* (Zhu et al. 2019; Wu et al. 2021). Historically, *Lonicera* has received extensive taxonomic evaluation and phylogenetic inference, while its phylogenetic relationships among sections, subsections, and species are still obscure (Theis et al. 2008; Nakaji et al. 2015).

*Lonicera angustifolia* var. *myrtillus* (Hook. f. & Thomson) Q. E. Yang, Landrein, Borosova & J. Osborne, a subalpine deciduous shrub with axillary pinkish white or purple-red paired flowers, occurs in shrubby hillside, sparse forests, and stony places along valleys with an altitude of 2400–4000 m. It belongs to the section *Isoxylosteum*, in which no complete plastome is reported so far. In order to better understand the molecular phylogenetic relationship of *Lonicera*, we reported and characterized the first complete chloroplast genome of *L. angustifolia* var. *myrtillus* using the next-generation sequencing technology. Furthermore, a phylogenomic analysis of this species and its relatives was also presented.

Fresh young leaves of *L. angustifolia* var. *myrtillus* were collected from Galongla Mountain, Bomi County, Xizang Autonomous Region (29.7643°N, 95.6975°E). Voucher specimen was preserved in PE (collector and collection number: Bing Liu (liubing@ibcas.ac.cn), Jian-Fei Ye & Hong-Qiang Xiao 3850). Genomic DNA extraction and next-generation sequencing were performed with an Illumina Hiseq platform by Shanghai OE Biotech. Co., Ltd. (http://oebiotech.bioon.com.cn/, Shanghai, China). We used Map function of Geneious R11 (Kearse et al. 2012) to select chloroplast reads using published chloroplast genome of *L. japonica* as reference (Kang et al. 2018). Then, these filtered reads were *de novo* assembled with Geneious R11. Gaps were filled using Fine Tuning function of Geneious R11. The assembled chloroplast sequence was then annotated using the Plann (Huang and Cronk 2015). Eventually, annotations were verified by Geneious R11.

The complete chloroplast genome sequence of *L. angustifolia* var. *myrtillus* is with 156,222 bp in length as a circle, which has a characteristic quadripartite structure with a large single-copy (LSC) region of 89,838 bp, a small single-copy (SSC) region of 19,211 bp, and a pair of inverted repeats (IRs) of 23,509 bp. In *L. angustifolia* var. *myrtillus* chloroplast genome, a total of 114 functional genes were identified, including 80 protein-coding genes (PCGs), 30 tRNA genes, and four rRNA genes. The total sequence GC content of the complete plastome is 38.4%.

Species phylogeny intra *Lonicera* and the phylogenetic position of *L. angustifolia* var. *myrtillus* were evaluated based on whole plastome data. The complete chloroplast genome sequences of *L. angustifolia* var. *myrtillus* and other 16 species from *Lonicera*, with *Heptacodium miconioides* and *Triosteum pinnatifidum* from Caprifoliaceae as outgroup were used for phylogenetic analysis. A total of 19 complete chloroplast genomes were aligned using MAFFT (Katoh et al. 2005). The phylogenetic tree was reconstructed and analyzed by the maximum-likelihood method with 1000 bootstrap values replicate at each node based on GTR + G model in RAxML software (Kozlov et al. 2019). The final tree was edited using the iTOL version 5.0 online web (https://itol.embl.de/) (Letunic and Bork 2016). A robust phylogeny of *Lonicera* was obtained based on the whole plastome data, and the genus was resolved as a monophyletic clade consisting two well supported clades, subgen. *Lonicera* and subgen. *Caprifolium* (Figure 1). Our result supports the classification of the two subgenera in *Lonicera* proposed by Rehder (1903, 1913), Hara (1983), and Hsu and Wang (1988) and coincides with previous molecular phylogenetic studies (Theis et al. 2008; Smith 2009; Nakaji et al. 2015; Zhu et al. 2019). However, all the four sections (sect. *Coeloxylosteum*, sect. *Isika*, sect. *Isoxylosteum*, and sect. *Nintooa*) classified in the subgen. *Lonicera* proposed by Rehder (1903, 1913) and Hsu and Wang (1988) were not supported as monophyly, all three of these sections are nested within the section *Isika* (Theis et al. 2008; Nakaji et al. 2015; Zhu et al. 2019), indicating that phylogenetic relationship intra subgen. *Lonicera* is complicated and not exactly consistent with traditional morphology. More species of this subgenus are needed to further investigate its phylogeny. As the first species whose whole plastome was reported in sect. *Isoxylosteum*, *L. angustifolia* var. *myrtillus* was indicated closely related to *L. nervosa* in a polyphyletic group, sect. *Isika,* which is consistent with previous study based on nuclear and chloroplast DNA sequences by Theis et al. (2008). Our results provide valuable data and shed light on the phylogenomic study of *Lonicera*.

**Figure 1.**
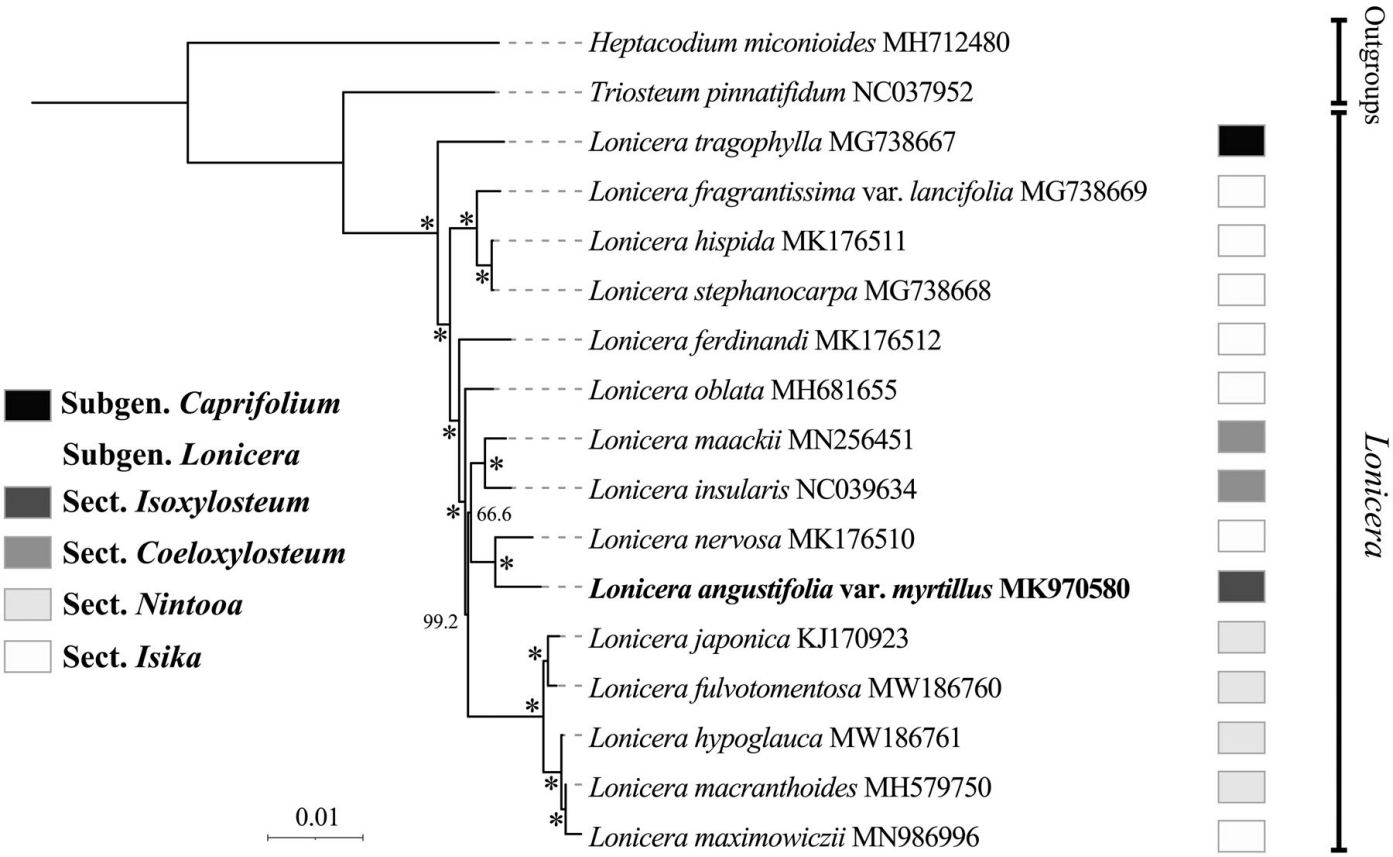
A phylogenetic tree of *Lonicera* L. inferred from the maximum-likelihood method based on the complete chloroplast genome data. *The clade support value of 100 that was generated from the maximum likelihood method. The taxonomic groups are following Rehder (1903, 1913), Hara (1983), and Hsu and Wang (1988).

## Data Availability

The genome sequence data that support the findings of this study are openly available in GenBank of NCBI at https://www.ncbi.nlm.nih.gov/ under the accession MK970580. Raw Illumina data were deposited in the NCBI Sequence Read Archive (SRA: SUB8882578, BioProject: PRJNA691677, and Bio-Sample: SAMN17302025).
